# Early Postnatal Pharmacological Intervention Rescues the Disruption of Developmental Connectivity in MAO-A KO Mice

**DOI:** 10.1007/s12264-024-01304-0

**Published:** 2024-10-24

**Authors:** Qian Xue, Hanpeng Xu, Muye Zhu, Bin Qian, Lei Gao, Lin Gou, Houri Hintiryan, Jean C. Shih, Hong-Wei Dong

**Affiliations:** 1 Department of Pharmacology and Pharmaceuticals Sciences, Mann School of Pharmacy, University of Southern California, Los Angeles, CA 90033 USA; 2 Department of Neurobiology, David Geffen School of Medicine, University California Los Angeles, Los Angeles, CA 90089 USA; 3 of Integrative Anatomical Sciences, Keck School of Medicine, University of Southern California, Los Angeles, CA 90032 USA

**Dear Editors,**

The term “developmental disconnection syndromes” (DDSs) was first coined by Geshwind and Levitt in 2007 [1] to describe the weakening of already formed connections or an absence of certain connections to establish correct organization *de novo* in early developmental stages. DDSs include a number of neuropsychiatric diseases, such as autistic spectrum disorder (ASD) [1, 2], which is characterized by impaired social communication and repetitive and stereotyped behaviors. Nevertheless, the exact etiology underlying these “disconnections” and their abnormal developmental trajectory remains largely unclear. Even less known is whether developmental disconnections relevant to autism can be rescued by early intervention thereby preventing neuropsychiatric and repetitive symptoms in later stages. This study addresses these important questions.

Serotonin, (5-hydroxytryptamine, 5-HT), plays important roles in cortical circuit formation and modulates neural plasticity during postnatal time periods critical for brain development [3–6]. For example, 5-HT functions as a trophic factor providing essential signals for targeting axons, dendritic growth, and synapse formation and establishing cortical circuits [4, 5]. 5-HT also affects oligodendrocyte development and myelination [7]. Thus, alteration of 5-HT levels at early developmental stages disrupts the normal wiring of the cerebral cortex, which in turn causes permanent perturbations of brain function and results in neuropsychiatric disorders [4, 8].

Monoamine oxidase (MAO; deaminating, EC 1.4.3.4) is a key enzyme involved in the degradation and regulation of monoamine neurotransmitters in both the brain and peripheral tissue [9]. The MAO-A isoenzyme preferentially targets 5-HT, norepinephrine, and dopamine as substrates, while MAO-B prefers phenylethyamine and benzylamine as substrates [9, 10]. MAO-A KO mice show elevated levels of brain 5-HT compared to wild-type (WT) mice, exhibiting a 9-fold increase at postnatal day 1 and 6-fold at day 12 [11]. These mice also exhibit autistic-like behaviors [9]. We hypothesized that PCPA (para-chloro-phenylalanine), which inhibits tryptophan hydroxylase, the rate-limiting enzyme for 5-HT synthesis and restores 5-HT levels to normal levels, may rescue the aberrant wiring and provide a molecular basis of disorders associated with disrupted 5-HT loads. Here, for the first time, we report that disruptions of neuronal connectivity can be rescued by pharmacological intervention during the early postnatal period using MAO-A KO mice as a model.

To test this hypothesis, we first quantified myelinated axons in the dorsal striatum [caudoputamen (CP)] in adolescent P42-day-old MAO-A KO mice and their WT littermates using a black-gold myelin staining method [12]. We focused on the CP given that significant reductions of cortico-striatal axons and aberrant wiring were previously shown in this structure in MAO-A/B KO mice [13]. Representative coronal sections through the CP were registered onto the corresponding levels of a standard mouse brain atlas—the Allen Reference Atlas [14]. Both large bundles and diffuse myelin fibers were quantified. Large myelin bundles called fascicles are composed of fiber tracts that traverse the striatum through the cortico-thalamic, thalamo-cortical, cortico-brainstem, and cortico-spinal tracts, while diffuse myelin fibers primarily comprise cortico-striatal and thalamo-striatal projection axons that innervate the CP. We found that, compared to WT controls, the numbers of both large and diffuse myelin bundles in MAO-A KO mice were significantly reduced to 76% and 75%, respectively (for SEM of percentage changes and *P* value see Table S1) (Fig. S1A–C).

Next, we demonstrated that the reduction of these myelinated axons occurred in the early developmental stage before puberty. At P10, no diffused myelin fibers, but only large bundles (Fig. S1D) were observed. At P15, myelinated axons and fiber bundles began to be visible in the CP of both WT and MAO-A KO mice. At this stage, the number of myelinated axons was too low to measure and there were no differences in fiber bundles between MAO-A KO and WT mice (Fig. S1D). We compared the numbers of myelinated axons in P28 mice (before puberty), which showed that diffuse myelin fibers (71% of WT) and the total number of fibers (88% of WT) were significantly reduced in MAO-A KO mice, while the large bundles did not differ between MAO-A KO and WT mice (Fig. S1D). These data suggest that disruptions of axonal trajectory and myelination in the CP of MAO-A KO mice occur during early postnatal developmental stages prior to puberty.

To investigate if the disruption of the axonal trajectory and myelination in the CP of MAO-A KO mice is preventable, we treated the KO mice with PCPA to reduce the 5-HT levels. MAO-A KO pups at the age of P1-P14 received daily injections of either PCPA (300 mg/kg/day, i.p.) or saline (Fig. 1A). Mice were sacrificed at P42 and brains were dissected and stained with black-gold to quantify CP myelinated axons and fiber bundles (fascicles). Consistent with the above results, the total number of diffuse myelinated fibers and large bundles displayed a trend of reduction in the CP of MAO-A KO mice injected with saline compared to that of WT mice (Fig. 1B, D). Because the CP is a large region with structural and functional heterogeneity, we subdivided it into 4 major divisions at the intermediate level, namely the dorsomedial (CPi.dm), dorsolateral (CPi.dl), ventrolateral (CPi.vl), and ventromedial (CPi.vm) CPi based on our previous work [13]. We then counted the myelinated bundles and axons in each of these divisions in all four experimental groups (Fig. 1C). As expected, our results showed significant reductions of myelinated axons in three of the four CP divisions (dorsolateral, ventrolateral, and ventromedial) in the saline-treated MAO-A KO mice compared with saline-treated WT mice (Fig. 1E, F, Table S1). However, following PCPA treatment, the reduction of diffuse myelin fibers in the ventrolateral CPi (82% of WT) and ventromedial CPi (86% of WT) were significantly reversed in MAO-A KO mice (Fig. 1B, E). Reductions of large bundles in the dorsolateral CPi (93% of WT) and ventrolateral CPi (79% of WT) in MAO-A KO mice were also rescued in the PCPA-treated group compared to the saline-treated MAO A-KO mice (Fig. 1B, F). Importantly, PCPA treatment did not affect the number of axons in WT mice (Fig. S2A-C).

**Fig. 1:**
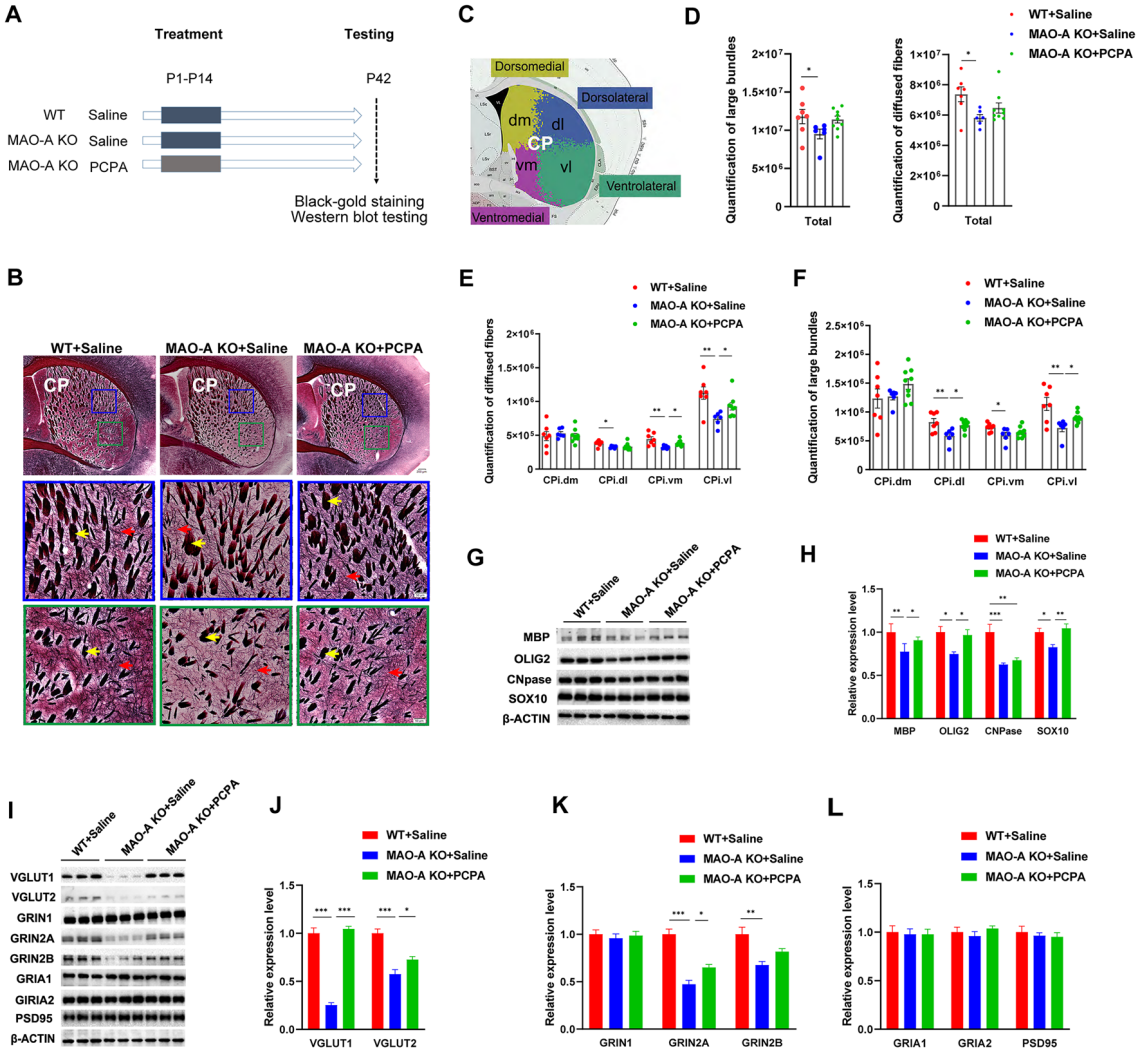
The disruption of developmental connectivity in the CP of MAO-A KO mice is rescued after early postnatal PCPA treatment. **A** Schematic of the experimental design. MAO-A KO mice received daily injections of either PCPA (300 mg/kg/day, i.p.) or saline at P1-P14. Age-matched WT littermates received daily injections of saline. Mice were sacrificed at P42 for black-gold staining and Western blot analyses. **B** Black-gold stained myelinated fibers in the caudoputamen (CP) of MAO-A KO mice treated with saline or PCPA and WT mice treated with saline. The magnified views of the boxed regions show the distinct appearance of large fiber bundles and diffuse myelinated fibers. Red arrows, diffuse myelinated fibers; yellow arrows, larger fascicles. CP: caudoputamen. Scale bars, upper panel, 200 μm, middle and lower panels, 50 μm. **C** The CP is subdivided into 4 divisions based on our previous work [13], namely the dorsolateral (CPi.dl), ventrolateral (CPi.vl), ventromedial (CPi.vm), and dorsomedial (CPi.dm) CPi (intermediate CP), to facilitate quantitative comparison of myelinated axons. **D–F** All experiments were performed in WT and MAO-A KO mice treated with saline and MAO-A KO mice treated with PCPA. **D** Quantification of large bundles and diffuse fibers in the total CP shows a significant reduction in both fiber types in saline-treated MAO-A KO mice compared to WT mice. **E** Quantification of the myelinated diffuse fibers in 4 divisions of the CP showing significant recovery of the fibers in the ventromedial and ventrolateral CPi in MAO-A KO mice following PCPA treatment. **F** Quantification of the large bundles in 4 divisions of the CP showing significant recovery of the fibers in the dorsolateral and ventrolateral CPi in MAO-A KO mice following PCPA treatment. Data are presented as the mean ± SEM. *n =* 7 for WT + Saline, *n =* 6 for MAO-A KO + Saline, *n =* 9 for MAO-A KO + PCPA, *P <0.05, **P <0.01. **G**, **H** Western blot analysis (G) and quantitation by densitometry (H) for MBP, OLIG2, CNPase, and SOX10 in the CP. **I** Western blot analysis for the glutamate transporters VGLUT1, VGLUT2, glutamate NMDA receptor subunits GRIN1, GRIN2A, GRIN2B, glutamate AMPA receptor subunits GRIA1, GRIA2, and the scaffolding protein PSD95 in the CP of MAO-A KO mice with saline or PCPA treatment and WT mice treated with saline. **J–L** Quantitation by densitometry for the glutamate transporters VGLUT1, VGLUT2 (J), the glutamate receptor GRIN1, GRIN2A, GRIN2B (K), the glutamate AMPA receptor GRIA1, GRIA2, and the scaffolding protein PSD95 (L) in the CP. All signals are normalized to β-actin. Data are presented as the mean ± SEM. *n =* 3, *P <0.05, **P <0.01, ***P <0.001.

To further elucidate how pharmacological manipulation of 5-HT levels in early postnatal stages affects the developmental myelination in the CP of MAO-A KO mice, we investigated the expression of myelin-related proteins in CP by applying Western blot analysis. The proteins included myelin basic protein (MBP), oligodendrocyte transcription factor (OLIG2), SRY-box transcription factor 10 (SOX10), and myelin-associated enzyme CNPase (Fig. 1G, H). The expression of all myelin proteins in the CP was significantly reduced in saline-treated MAO-A KO mice compared to saline-treated WT mice. Specifically, the expression of MBP, OLIG2, and SOX10 in saline-treated MAO-A KO mice was 76%, 75%, and 83% of the WT saline treatment group (Fig. 1G, H). However, PCPA administration rescued the expression of MBP, OLIG2, and SOX10 to levels comparable to WT controls (91%, 97%, and 104% of WT), without affecting the reduction of CNPase (63% of WT, Fig. 1G, H). PCPA treatment did not alter the expression levels of these myelin proteins in WT control mice (Fig. S2D, E). Together, this evidence supports the hypothesis that early PCPA administration reverses or rescues, at least partially, the reduction of myelination in the striatum in MAO-A KO mice.

Next, we characterized the potential disruption of synaptic connectivity in MAO-A KO mice and determined whether this too could be rescued by PCPA administration in early development. We measured the expression of two presynaptic proteins: vesicular glutamate transporter 1 (VGLUT1), which is expressed in the presynaptic terminals of cortico-striatal axons, and vesicular glutamate transporter 2 (VGLUT2), which is expressed in the presynaptic terminals of thalamo-striatal axons. The expression of VGLUT1 and VGLUT2 in saline-treated MAO-A KO mice was significantly reduced to 25% and 58% of the WT, respectively (Fig. 1I, J). However, after PCPA treatment, the VGLUT1 expression in MAO-A KO CP was significantly increased to ~105%, while VGLUT2 expression was partially rescued (73% of WT) (Fig. 1I, J). Compared to the MAO-A KO saline group, these increments in the MAO-A KO PCPA treatment group were significant. PCPA treatment in WT mice did not produce significant differences compared to saline-treated WT mice (Fig. S2F, G). This evidence suggests that the disrupted expression of presynaptic proteins in both cortico-striatal and thalamo-striatal axonal terminals in MAO-A KO mice can be restored *via* PCPA treatment during early development.

Finally, we measured the expression levels of glutamate receptor proteins, which are expressed post-synaptically in striatal neurons. Our data showed that in MAO-A KO mice, the expression levels of two N-methyl-D-aspartate (NMDA) receptors, GRIN2A and GRIN2B, in the CP were significantly reduced (47% and 68% of WT; Fig. 1I, K). PCPA administration was able to rescue GRIN2A expression to levels comparable to WT controls (65% of WT), but PCPA administration did not significantly rescue GRIN2B expression (82% of WT). The expression levels of GRIN1 in the CP did not significantly differ between MAO-A KO mice and WT mice (96% of WT), and administration of PCPA did not affect their expression levels (99% of WT; Fig. 1I, K). In addition, our data showed that the expression levels of the glutamate AMPA receptors, GRIA1 and GRIA2, as well as another postsynaptic protein, PSD95, in the CP did not significantly differ between MAO-A KO mice and WT mice with saline treatment (Fig. 1I, L), but PCPA administration appeared to reduce GRIA1 receptors in WT mice (75% of WT; Fig. S2F, G).

Taken together, we demonstrated that MAO-A KO mice displayed disruptions of neuronal connectivity (or “disconnections”) in several ways: (1) reduction of striatal myelinated axons and myelin-related proteins (MBP, OLIG2, and SOX10); (2) reduction of the presynaptic proteins VGLUT1 and VGLUT2, which are expressed in cortico-striatal and thalamo-striatal axonal terminals, respectively; and (3) selective reduction of the postsynaptic proteins GRIN2A and GRIN2B, but not GRIN1 or glutamate AMPA receptors. Consistent with these results, our previous study also showed morphological changes (i.e., dendritic arbors) of cortical pyramidal neurons in MAO-A KO mice [15], which presumably generate cortico-striatal projections. However, upon early postnatal (P1 through P14) treatment with PCPA all of these connectivity disruptions were partially rescued or reversed to the level of WT animals in later developmental stages (P42).

This finding is consistent with our previous work [16], which showed that early postnatal inhibition of 5-HT by PCPA in MAO-A KO mice leads to a significant and long-lasting reduction in 5-HT levels in the forebrain, with these neurochemical changes persisting into adulthood. The same study also found that PCPA treatment significantly reduces the perseverative behavior and anxiety typically displayed by MAO-A KO mice. The present study indicates that behavioral abnormalities in adult MAO-A KO mice (and presumably in humans with MAO-A deficiency) are a consequence of disruption in neural circuits during early postnatal development, but are “preventable” or “reversible” with early pharmacological interventions that lower their neural 5-HT levels. This is the first study to demonstrate that connectivity disruption can be pharmacologically rescued, at least partially, by reducing the abnormally high levels of neural 5-HT in the early postnatal development of MAO-A KO mice. This effect appeared to be long-term or even permanent for restoring brain behavior and function.

Approximately 30−50% of ASD patients exhibit hyperserotonemia, with males more likely to have this condition than females among pre-pubertal children [17]. High levels of 5-HT in autistic children are generally associated with more intense symptoms [18]. ASD has also been linked to genetic polymorphisms in the 5-HT system [19, 20]. Consequently, MAO-A KO mice serve as a robust model to investigate how manipulating 5-HT levels during early postnatal stages affects the development of cortical neural circuits. Our previous study showed that MAO-A KO mice exhibit neuropathological alterations reminiscent of typical autistic features, including reduced thickness of the corpus callosum, increased arborization of apical dendrites, reduction in the number and length of basilar dendritic branches of pyramidal projection neurons in the orbitofrontal cortex, and disrupted microarchitecture of the cerebellum [21].

In this study, we demonstrated that pharmacologically reducing abnormally high 5-HT levels during a critical window for the formation of circuit wiring in the early postnatal stage of MAO-A KO mice, at least partially, prevents or restores the disrupted connectivity. Therefore, MAO-A KO mice provide a model to investigate how changes in 5-HT levels during key postnatal developmental stages affects cortical wiring and how these cortical disconnections lead to long-term alterations in brain function and behavior. Moreover, this study offers key insights into novel and practical strategies for early intervention and prevention, potentially decelerating, diminishing, or even preventing the effects of developmental disconnection syndromes in adulthood.

## Supplementary Information

Below is the link to the electronic supplementary material.

Supplementary file1 (PDF 908 kb)
