# Supplementary material for: Early Postnatal Pharmacological Intervention Rescues the Disruption of Developmental Connectivity in MAO-A KO Mice
Source: Neurosci Bull. 2024 Oct 24;41(2):339–43. doi: 10.1007/s12264-024-01304-0 (PMC11794938; doi:10.1007/s12264-024-01304-0)
Supplement: Supplementary file 1 — Supplementary file1 (PDF 908 kb) [file 12264_2024_1304_MOESM1_ESM.pdf]

Supplementary Materials

Table S1. Myelin fibers and proteins in CP of mice treated with PCPA or Saline

Measure fibers/proteins	WT (SEM of percentage changes)	MAO-A KO (SEM of percentage changes)	WT+Saline (SEM of percentage changes)	MAO-A KO +Saline (SEM of percentage changes)	MAO-A KO + PCPA (SEM of percentage changes)	*P* value (WT vs MAO-A KO)	*P* value (WT+Saline vs MAO-A KO+Saline)	*P* value (MAO-A KO+Saline vs MAO A KO+PCPA)	Figure
**Large bundles**	100% (0.062)	76% (0.089)				0.0432			S1C
**Diffuse myelin fibers**	100% (0.095)	75% (0.028)				0.0396			S1C
**Diffuse myelin fibers (P28)**	100% （0.054）	71% （0.072）				0.0009			S1D
**Total CP bundles (P28)**	100% （0.026）	88% （0.029）				0.0106			S1D
**Total CP bundles**			100% （0.079）	81% （0.054）	97% （0.038）		0.0371	0.0645	1D
**Total diffuse CP fibers**			100% (0.066)	79% (0.030)	88% (0.045)		0.0121	0.2364	1D
**Diffuse myelin CPi.dl**			100% (0.042)	86% (0.019)	88% (0.043)		0.0309	0.7512	1E
**Diffuse myelin CPi.vm**			100% (0.077)	73% (0.021)	86% (0.032)		0.0016	0.0113	1E
**Diffuse myelin CPi.vl**			100% (0.082)	66% (0.037)	82% (0.048)		0.0018	0.0495	1E
**Large bundles CPi.dl**			100% (0.076)	71% (0.063)	93% (0.035)		0.0031	0.0129	1F
**Large bundles CPi.vm**			100% (0.038)	82% (0.068)	89% (0.042)		0.0202	0.3246	1F

**Large bundles CPi.vl**			100%<br>(0.039)	63%<br>(0.100)	79%<br>(0.049)		0.0036	0.0441	1F
**MBP**			100%<br>(0.097)	76%<br>(0.092)	91%<br>(0.038)		0.0017	0.0172	1H
**OLIG2**			100%<br>(0.065)	75%<br>(0.026)	97%<br>(0.062)		0.0140	0.0297	1H
**CNPase**			100%<br>(0.091)	63%<br>(0.018)	68%<br>(0.027)		0.0004	0.2031	1H
**SOX10**			100%<br>(0.044)	83%<br>(0.030)	104%<br>(0.052)		0.0217	0.0058	1H
**VGLUT1**			100%<br>(0.057)	25%<br>(0.025)	105%<br>(0.027)		<0.0001	<0.0001	1J
**VGLUT2**			100%<br>(0.046)	58%<br>(0.046)	73%<br>(0.030)		<0.0001	0.0436	1J
**GRIN2A**			100%<br>(0.056)	47%<br>(0.041)	65%<br>(0.032)		<0.0001	0.0189	1K
**GRIN2B**			100%<br>(0.076)	68%<br>(0.037)	82%<br>(0.032)		0.0015	0.1117	1K

Supplementary Figures

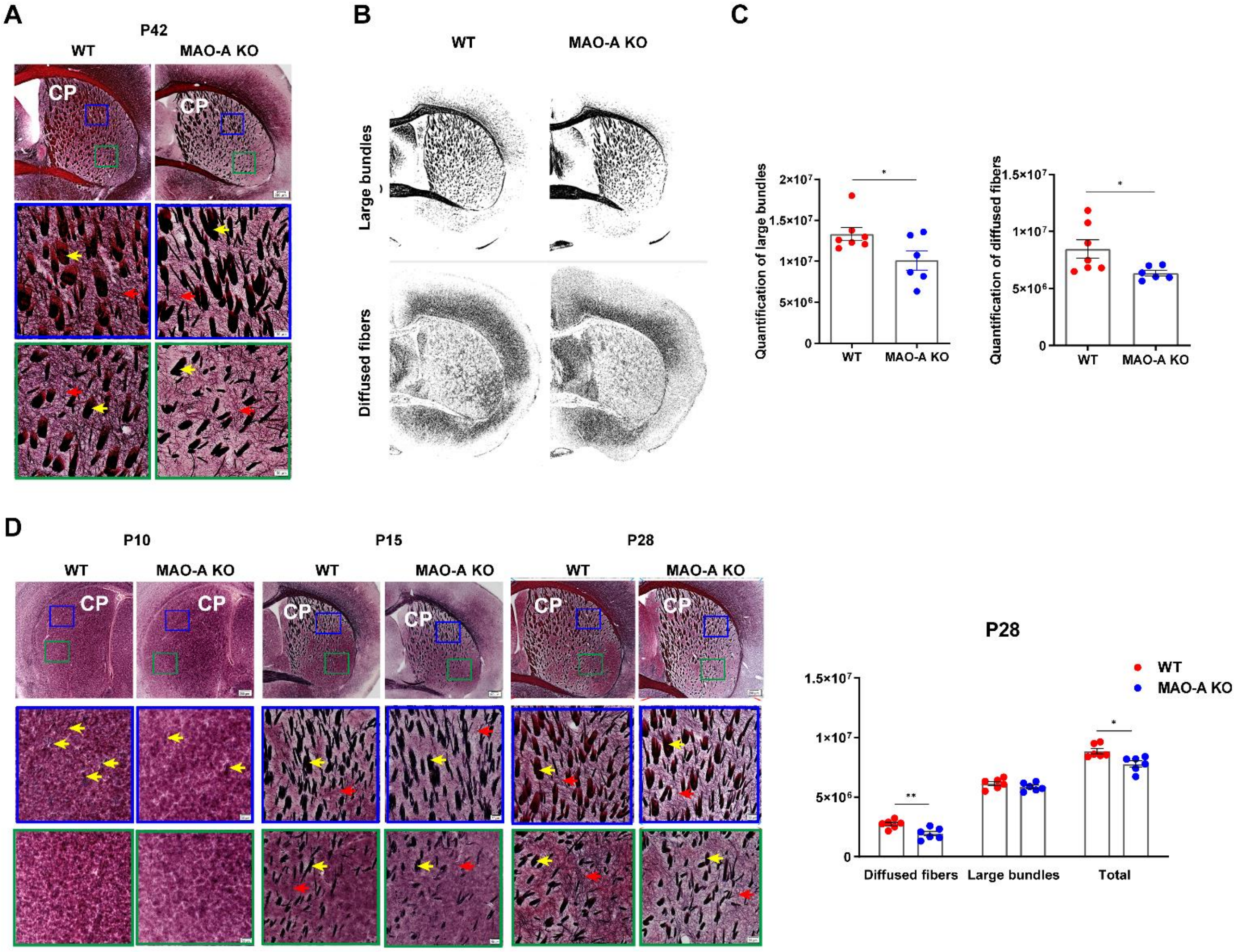

Fig. S1. Reduction of myelin in the CP of MAO-A KO mice.

A Myelinated fibers stained with black-gold in the dorsal striatum (CP in rodents) of WT and MAO-A KO mice. The magnified views of the boxed regions show distinctive large fiber bundles (yellow arrowheads) and individual myelinated axons (red arrowheads). Scale bars, upper panel, 200 μm, middle and lower panels, 50 μm. **B** Using informatics tools, these myelinated fibers are separated and filtered into large fiber bundles (upper) and diffuse individual axons (lower) in the CP of WT and MAO-A KO mice, which then were used for quantitative comparison. **C** The quantification of the myelinated large bundles and diffuse fibers in the total CP of MAO-A KO and WT mice. Data are presented as the mean ± SEM. *n* = 7 for WT, *n* = 6 for MAO-A KO, **P* <0.05. **D** Myelinated fibers in the CP of MAO-A KO mice and WT mice stained with black-gold at P10, P15, and P28. Myelin bundles and axons are not fully developed at P10 and P15. Scale bars, upper panel, 200 μm, middle and lower panels, 50 μm. Bar graphs showing the quantification of the myelinated diffused fibers, large bundles, and total fibers in the CP of MAO-A KO mice and WT mice at P28. Data are presented as the mean ± SEM. *n* = 6 for WT, *n* = 6 for MAO-A KO, **P* <0.05, ***P* <0.01.

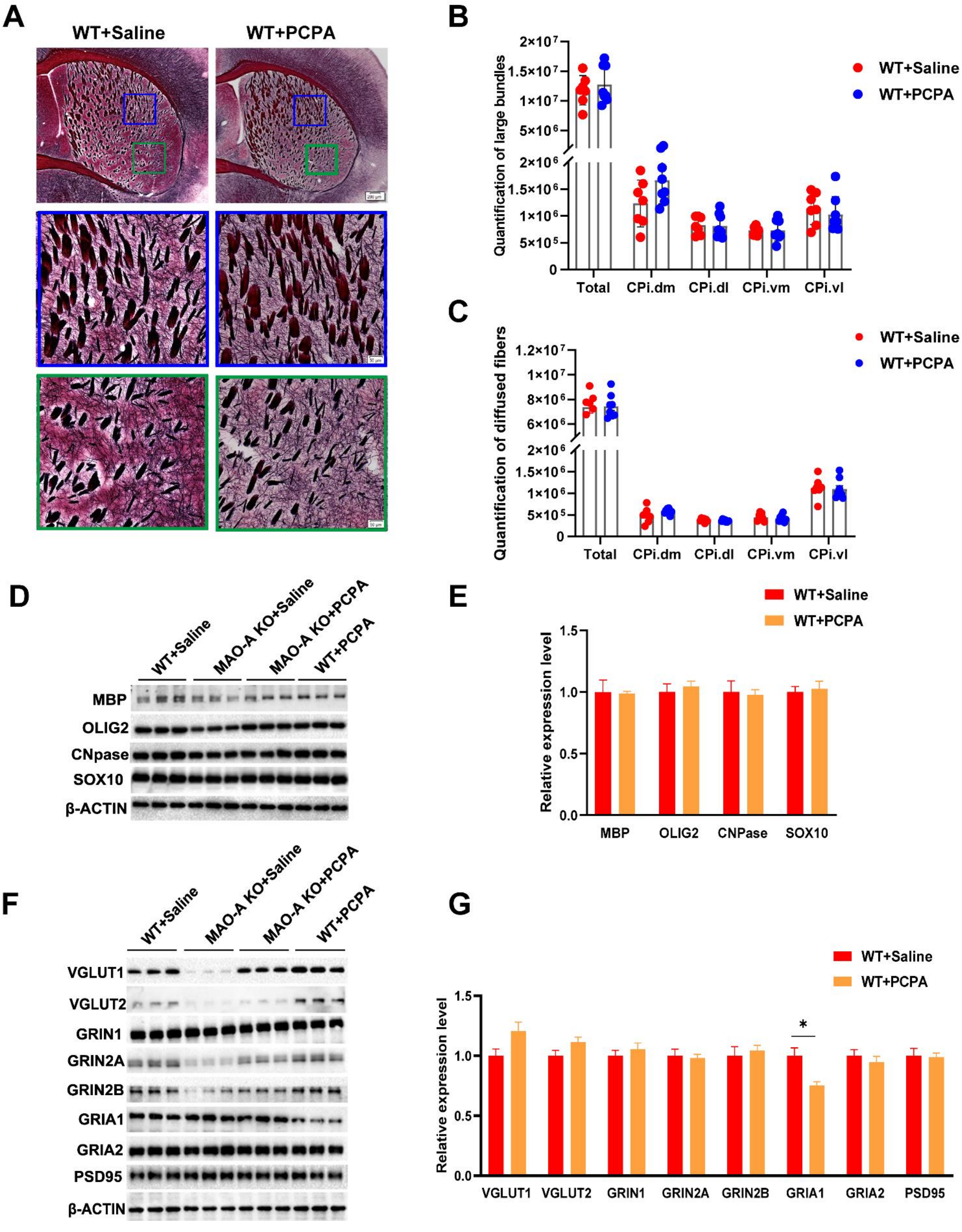
A
WT+Saline
WT+PCPA
B
Quantification of large bundles
WT+Saline
WT+PCPA
Total
CPi.dm
CPi.dl
CPi.vm
CPi.vl
C
Quantification of diffused fibers
WT+Saline
WT+PCPA
Total
CPi.dm
CPi.dl
CPi.vm
CPi.vl
D
WT+Saline
MAO-A KO+Saline
MAO-A KO+PCPA
WT+PCPA
MBP
OLIG2
CNpase
SOX10
β-ACTIN
E
WT+Saline
WT+PCPA
Relative expression level
MBP
OLIG2
CNPase
SOX10
F
WT+Saline
MAO-A KO+Saline
MAO-A KO+PCPA
WT+PCPA
VGLUT1
VGLUT2
GRIN1
GRIN2A
GRIN2B
GRIA1
GRIA2
PSD95
β-ACTIN
G
WT+Saline
WT+PCPA
Relative expression level
VGLUT1
VGLUT2
GRIN1
GRIN2A
GRIN2B
GRIA1
GRIA2
PSD95

Fig. S2. PCPA treatment in WT mice does not alter myelin and synaptic protein levels in the CP.

A Black-gold-stained myelinated fibers in the CP of WT mice with saline or PCPA treatment. Scale bars, upper lane, 200 μm, middle and lower lanes, 50 μm. The left panels have the same data as the left panels of Figure 1B. **B** Quantification of the large bundles in total CP, dorsomedial (CPi.dm), dorsolateral (CPi.dl), ventromedial (CPi.vm), and ventrolateral (CPi.vl) of WT mice treated with PCPA or saline (see Fig. 1C for CP subdivisions). **C** Quantification of diffuse fibers in the total CP, CPi.dm, CPi.dl, CPi.vm, and CPi.vl of WT mice treated with PCPA or saline. Data are presented as the mean ± SEM. n = 7 for WT + saline, n = 8 for WT + PCPA. **D** Western blots for MBP, OLIG2, CNPase, and SOX10 in the CP of MAO-A KO and WT mice treated with PCPA or saline. The left 9 lanes are the same data as in Fig. 1G. **E** Quantitation by densitometry for MBP, OLIG2, CNPase, and SOX10 in the CP of WT mice treated with PCPA or saline. **F** Western blots for the glutamate transporters VGLUT1, VGLUT2, glutamate NMDA receptor subunits GRIN1, GRIN2A, GRIN2B, glutamate AMPA receptor subunits GRIA1, GRIA2, and the scaffolding protein PSD95 in the CP of MAO-A KO and WT mice with saline or PCPA treatment. The left 9 lanes are the same data in the figure. **G** Analysis by densitometry for the glutamate transporters VGLUT1, VGLUT2, glutamate NMDA receptor subunits GRIN1, GRIN2A, GRIN2B, glutamate AMPA receptor subunits GRIA1, GRIA2, and the scaffolding protein PSD95 in the CP of WT mice with saline or PCPA treatment. Data are presented as the mean ± SEM. n = 3, *P <0.05.

Methods and Materials

Animals

Male MAO-A (A863T) KO and male wild-type (WT) mice in the C57 BL/6 strain were generated and genotyped as previously described [1]. Briefly, genomic DNA from tail samples and Taq polymerase (Invitrogen, Carlsbad, CA, USA) were used for PCR. To identify MAO-A, the following primers were used: MAO-A forward: 5′-ACGCGCTCTTCTGGTGCAT-3′, MAO-A reverse: 5′-AGCTTACTTCAGGGC-3′. MAO-A PCR products were then digested with Dra1 (New England Biolabs, Ipswich, MA, USA) and run on the gels. MAO-KO mice with an A→T point mutation, lost the Dra 1 cutting site, while WT mice showed two Dra 1 cutting bands.

All animal experiments were performed in accordance with the regulations of the National Institutes of Health Guide for the Care and Use of Laboratory Animals and followed the institutional guidelines set by the Animal Care and Use Committee at USC and the Animal Research Committee at the University of California, Los Angeles. Animals were maintained in a light-controlled animal facility (12-h light and 12-h dark cycle) at 21–22°C and 51% humidity. All animals had *ad libitum* access to food and water.

PCPA injection

Male MAO-A KO and male WT pups received either PCPA (300 mg/kg per day, within an injection volume of 10 mL/kg, intraperitoneal) or the same volume of vector solution (0.09% saline) from Postnatal day 1 to day 14 at 10:00 every day (n = 7 for WT + Saline, n = 6 for MAO-A KO + Saline, n = 9 for MAO-A KO + PCPA). Additional WT pups (n = 8) also received daily injections of PCPA (WT + PCPA). After this procedure, all animals were maintained until P42 after which they were transcardially perfused. Brains were dissected for black-gold staining and Western blot analysis as described below.

Black-gold staining

Animals were anesthetized with sodium pentobarbital, and then transcardially perfused with ~50 ml of 0.9% NaCl followed by 50 mL of 4% paraformaldehyde (PFA; pH 9.5). The brains were post-fixed in 4% PFA overnight at 4°C, after which they were embedded in 3% Type I-B agarose (Sigma-Aldrich) before sectioning. Four series of coronal sections were cut at 50-μm on a Compresstome (VF-700, Precision Instruments). One of four series of sections was mounted on slides in PBS buffer and dried at room temperature for 48-72 h.

Black-gold II powder (Histo-Chem, Inc.) was dissolved in 0.9% NaCl to obtain a final staining solution of 0.3%. The black-gold II staining solution was pre-heated and maintained at 60–65°C. Tissue sections mounted on slides were immersed in double-distilled water for 2 min and then transferred to the pre-heated black-gold II solution. Slides were incubated in this solution for 18–25 min, then rinsed in double-distilled water for 2 min. The sections were dehydrated in serial ethanols at 50%, 70%, 75%, 80%, 85%, 90%, 95%, and 100%, twice per concentration for 2 min. Sections were cleared in xylene and coverslipped with Permount (Sigma, 44581).

Image processing and informatics workflow for quantification

All sections were scanned under an Olympus VS120 microscope with a 10× air objective lens. Black-gold images were directly converted to tiff format prior to registration. The thresholded images that were used for the quantification of the data (discussed below) were not affected by changes in image contrast and brightness.

The black-gold-stained fiber signals were extracted and processed as described previously [2]. Briefly, the signal-to-noise ratio of the black-gold-stained images was improved by local adaptive mapping as

described previously [3]. For a normalized image $f(x,y) \in [0,1]$, an initial intensity mapping T is defined as

$$T(f(x,y),p) = sin^2\left(\frac{\pi}{2}f(x,y)^p\right), \quad p > 0$$

Using the first-order Taylor expansion approximation of $sin\ (u)$, the mapping is rewritten as

$$T(f(x,y),c) = \frac{\pi^2}{4}f(x,y)^c, \quad c = 2p$$

The mapping argument c is defined as $c = c_1 \cdot \frac{f_g(x,y)+\epsilon}{(1-f_g(x,y))+\epsilon} + c_2$, where $f_g(x,y) = f(x,y) * g(x,y)$ denotes the convolution between $f(x,y)$ and a Gaussian kernel $g(x,y)$, while c_1, c_2 are user-specified values. Locally adaptive contrast enhancement is achieved as follows:

$$E(x,y) = \frac{f(x,y)}{f_g(x,y)}T(f(x,y),c) + \frac{f(x,y)}{f_g(x,y)}\frac{\partial T(f(x,y),c)}{\partial c} \cdot (f(x,y) - f_g(x,y))$$

The mapping separates black-gold-stained fibers from the tissue background further apart in intensity. A scale parameter σ_L is selected such that a much greater response results from the convolution $E_{\sigma_L} = E * g(\sigma_L)$ at pixel locations corresponding to larger fiber bundles than those of diffuse axons. The large fibers are subsequently extracted by thresholding E_{σ_L}, while small fibers are obtained by thresholding E and further excluding pixel locations already determined as large fibers. Quantitative analysis of axonal densities in the CP followed the same method as described in our earlier publication [4].

Western blots

Male MAO-A KO and male WT mice treated with PCPA or saline were used for western blot analyses (*n* = 3 per group). Fresh striatal tissue samples from the brain slices were dissected on ice under an anatomical microscope. Each sample was lysed in RIPA Lysis and Extraction Buffer (ThermoFisher Scientific, 89900) and incubated on ice for 30 min. The samples were then centrifuged, supernatants collected, and protein concentrations determined by the BCA method. The same amount of protein in each sample were separated on 12% SDS-PAGE gels at a constant 100 mV voltage and transferred to polyvinylidene difluoride (PVDF) membranes at 300 mV for 1–3 h. The PVDF membranes were blocked by 3% BSA for 1 h, incubated overnight at 4°C with the primary antibodies MBP (1:2,000, Abcam, ab209328), OLIG2 (1:2,000, Abcam, ab109186), CNPase (1:2,000, ThermoFisher Scientific, PA5-27972), SOX10 (1:2,000, Abcam, ab155279), VGLUT1 (1:1,000, Abcam, ab180188), VGLUT2 1:200, Abcam, ab84103), GRIN1 (1:1,000, Abcam, ab17345), GRIN2A (1:1,000, Millipore, 07-632), GRIN2B (1:1,000,

Millipore, 06-600), GRIA1 (1:1,000, NeuroMab, 75-327), GRIA2 (1:1,000, NeuroMab, 75-002), PSD95 (1:1,000, BD Biosciences, 610495), or β-actin (1:4,000, Sigma-Aldrich, A2228), washed three times in TBS with 0.1% Tween-20, and then incubated in the related secondary antibodies conjugated with horseradish peroxidase (Sigma-Aldrich) for 1–2 h at room temperature. Immunoreactive bands were visualized using an enhanced chemiluminescence kit on a Bio-Rad Image Lab system. Densitometric analysis was done using ImageJ software. All experiments described in this study were independently repeated a minimum of three times.

Statistical analysis

All statistical analyses of all experimental data were performed using GraphPad Prism 8.0 (GraphPad) and are presented as the group mean ± SEM. Two groups were compared using independent two-tailed Student's *t*-tests. One-way ANOVA and Kruskal-Wallis tests followed by Benjamini-Hochberg multiple comparisons were applied for comparisons among three or more groups.

Data availability

The data that support the findings of this study are available from the corresponding author upon request. The datasets generated and/or analyzed during the current study will be available at http://brain.neurobio.ucla.edu/publications/.

Code availability

An in-house software, Connection Lens, was used to register (warp), threshold (segment), and annotate the labelling in all image data used in the striatal output analysis. This software has not been publicly released yet but will be available from the corresponding author upon request.

The study is reported in accordance with ARRIVE guidelines (https://arriveguidelines.org).

References and Notes

[1] Scott AL, Bortolato M, Chen K, Shih JC. Novel monoamine oxidase A knock out mice with human-like spontaneous mutation. Neuroreport 2008, 19: 739–743.

[2] Schmued L, Bowyer J, Cozart M, Heard D, Binienda Z, Paule M. Introducing Black-Gold II, a highly soluble gold phosphate complex with several unique advantages for the histochemical localization of myelin. Brain Res 2008, 1229: 210–217.

[3] Zhou Z, Sang N, Hu X. A parallel nonlinear adaptive enhancement algorithm for low- or high-intensity color images. EURASIP J Adv Signal Process 2014, 2014: 70.

[4] Hintiryan H, Foster NN, Bowman I, Bay M, Song MY, Gou L, *et al*. The mouse cortico-striatal projectome. Nat Neurosci 2016, 19: 1100–1114.
